# Isolation and Characterisation of Bacteriophage Selective for Key *Acinetobacter baumannii* Capsule Chemotypes

**DOI:** 10.3390/ph15040443

**Published:** 2022-04-02

**Authors:** Rosesathorn Soontarach, Potjanee Srimanote, Mark C. Enright, George Blundell-Hunter, Matthew J. Dorman, Nicholas R. Thomson, Peter W. Taylor, Supayang P. Voravuthikunchai

**Affiliations:** 1 Division of Biological Science, Faculty of Science and Natural Product Research Center of Excellence, Prince of Songkla University, Songkhla 90110, Thailand; rosesathron_pim@hotmail.com; 2 Center of Antimicrobial Biomaterial Innovation-Southeast Asia, Prince of Songkla University, Songkhla 90110, Thailand; 3 Graduate Program, Faculty of Allied Health Sciences, Thammasat University, Pathum Thani 12121, Thailand; psrimanote01@yahoo.com.au; 4 Department of Life Sciences, Manchester Metropolitan University, Chester Street, Manchester M1 5GD, UK; m.enright@mmu.ac.uk; 5 School of Pharmacy, University College London, London WC1N 1AX, UK; g.blundell-hunter@ucl.ac.uk (G.B.-H.); peter.taylor@ucl.ac.uk (P.W.T.); 6 Wellcome Sanger Institute, Wellcome Genome Campus, Hinxton, Cambridgeshire CB10 1SA, UK; md25@sanger.ac.uk (M.J.D.); nrt@sanger.ac.uk (N.R.T.); 7 Department of Infection Biology, Faculty of Infectious and Tropical Diseases, London School of Hygiene and Tropical Medicine, London WC1E 7HT, UK

**Keywords:** *Acinetobacter baumannii*, capsular type, bacteriophage, antibacterial agent, Podoviridae

## Abstract

Nineteen bacteriophages against five main capsular types of multidrug-resistant *Acinetobacter baumannii* were isolated from tertiary care hospital sewage. Eight representative phages from each capsular type were characterized and tested for their biological properties. The biological features revealed that phages T1245, T444, and T515 had a large burst size of more than 420 pfu/mL, together with a short latent period lasting less than 6 min, and were readily adsorbed to a bacterial host within 10 min. Moreover, these phages demonstrated host specificity and stability over a broad range of temperatures (−20 to 60 °C) and pH (5.0–9.0). A whole-genome analysis of six lytic and two temperate phages revealed high genomic similarity with double-stranded DNA between 40 and 50 kb and G + C content of 38–39%. The protein compositions disclosed the absence of toxin-coding genes. The phylogenic results, together with morphological micrographs, confirmed that three selected phages (T1245, T444, and T515) belong to the Podoviridae family within the order Caudovirales. The biological data and bioinformatics analysis indicated that these novel *A. baumannii* phages possess important enzymes, including depolymerase and endolysin, which could be further developed as promising alternative antibacterial agents to control *A. baumannii* infections.

## 1. Introduction

*Acinetobacter baumannii* is an emerging opportunistic Gram-negative pathogen that is associated with frequent and severe infections in the immunocompromised and in patients with prolonged hospitalization or long-term exposure to antimicrobials. The high prevalence and incidence of drug-resistant strains of *A. baumannii* in both developing and developed countries have been increasingly reported [1,2]. The growing resistance of *A. baumannii* to antimicrobial treatment is a serious challenge, causing adverse clinical effects associated with high economic costs [3]. Some strains of *A*. *baumannii* invariably produce capsular polysaccharides, which promote survival and adaptation to various environmental conditions, and may contribute to antimicrobial resistance and the evasion of host immune defenses [4]. K units are assembled on the lipid carrier molecule for growing sugar chains. The genes controlling the Gtr enzyme, Wzx translocase, Wzy polymerase, Wza, Wzb, and Wzc transport proteins play an important role in capsular polysaccharide (CPS) synthesis. Genetic sequence analysis identified over 100 predicted capsular loci (KL) in *A. baumannii* isolates. The chemical structures, such as the sugar compositions, length of the structure, linkage within and between the K units, and location of glycosidic bonds, have been used to confirm the KL types [5]. Of these 100 capsule types, the most common three are KL6, KL10, and KL47, which altogether occur in more than 10% of all nosocomial infections [6]. 

Nosocomial carbapenem-resistant *A. baumannii* (CRAB) infection has been reported as the most increasingly common opportunistic infection in tertiary care hospitals across Thailand [7,8]. The CDC report on antibiotic resistance in the United States listed CRAB as an urgent public health threat [9]. Genome analysis has revealed that the majority of CRAB strains correspond to the sequence type 2 (ST2) [10], which is the most common ST type of the global clone 2 (GC2) in European clones [11]. However, we reported no correlation between sequence types and KL types [6]. The management of infections caused by multidrug-resistant *A. baumannii* has relied on the use of the last-line polymyxin antibiotic colistin. However, the recent emergence of resistance, due to the modification of lipid A and the significant toxicity associated with colistin, has made the clinical use of this agent more problematic [12]. New antibacterial agents and alternative therapeutic paradigms are urgently needed for the treatment of multidrug-resistant (MDR) *A. baumannii* infections. 

Bacteriophages are natural antibacterial agents and are commonly isolated from environmental sources, such as water and soil. The most prevalent phages on earth are members of the order Caudovirales, which are the tailed phages with the larger genome in size of the dsDNA phage [13]. The first successful use of phage therapy in human patients with severe generalized *A. baumannii* infections was reported in 2017 [14]. Recent studies have suggested that phage may have therapeutic efficacy in vivo against MDR *A. baumannii* infections [15]. Polyvalent phage cocktails containing multiple phages have been developed for the treatment of bacterial infections [16] but these could be compromised by the interactions between phages and the human immune system, leading to an inappropriate inflammatory response that may be detrimental to the patient. Thus, to avoid such interactions, the properties of phage (physical characteristics, dose, purity, and route of administration) should be carefully examined before any proposals for clinical evaluation are advanced [17]. In addition, the identification of a receptor on bacterial surfaces involved in phage adsorption should be included [18]. Moreover, the proteins encoded by phages, such as depolymerases and endolysins, have been investigated for their capacity to act as alternative antibacterial therapies [19,20,21]. 

In this study, we report on the properties of phages isolated from wastewater at three major tertiary care hospitals from different locations in Thailand against MDR *A. baumannii* isolates. The phages were evaluated for biological properties in accordance with the criteria considered for potential therapeutic phage, including morphology, host range, burst size, and stability. In addition, the whole-genome annotation of phages was investigated, which provides vital information for further therapeutics development and applications.

## 2. Results

### 2.1. Phage Isolation, Purification, and Plaque Characterisation

**Figure 1:**
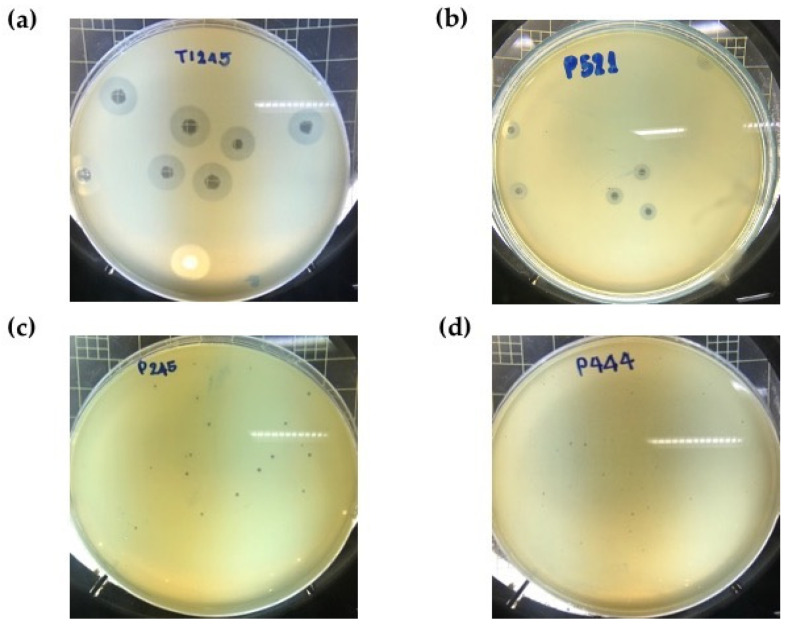
Plaque morphology of phages on different hospital isolates of multidrug-resistant *A. baumannii.* (**a**) Big and bright plaque (8–10 mm) on KL10 isolate, (**b**) medium-size and bright plaque (5–7 mm) on KL10 isolate, (**c**) small and slightly dim plaque (2–4 mm) on KL6 isolate, and (**d**) extremely small and slightly dim plaque (0.5–1 mm) on KL52 isolate.

A total of twenty phage selective for MDR *A. baumannii* were isolated from sewage water samples derived from major national referral hospitals in Thailand (Supplementary Table S1). After incubation at 37 °C for 12 h with different primary host strains, lytic phages formed clear plaques with different morphology and zones of lysis between 0.5 and 10.0 mm in diameter (Figure 1). The plaques from some phages, such as P515, were surrounded by translucent halos that increased in diameter over the time of incubation while the plaque size remained constant (Supplementary Figure S1), indicating the production of polysaccharide depolymerases [22].

### 2.2. Determination of Phage Host Range

**Figure 2:**
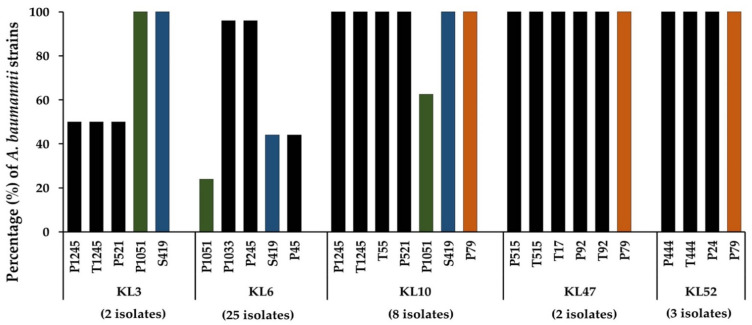
Lytic activity of 18 phages on 49 multidrug-resistant *A. baumannii* clinical isolates of capsular types KL3, KL6, KL10, KL47, and KL52. Phage lysates were spotted onto the lawns formed by *A. baumannii*. Phages P1051, S419, and P79 were able to infect bacteria with three distinct capsular types. Three different color bars indicate phages P1051, S419, and P79 that could infect three capsular types.

In this study, the lytic spectrum was determined by spot tests onto the lawns formed by 49 strains of MDR *A. baumannii* belonging to the most common capsular types (KL2, KL3, KL6, KL10, KL47, KL49, and KL52) (Supplementary Table S2). Our results further confirmed that most of the *A. baumannii* phages were host-specific; thus, they could infect or lyse only a single or limited number of different capsular types (Figure 2). However, plaque formation on the lawn formed by five capsular types (KL3, KL6, KL10, KL47, and KL52) were detected. Some phages demonstrated almost 100% lytic activity, including P1033 and P245 on KL6 strains, P1245, T1245, T55, P521, S419, and P79 on KL10 strains, T17, P92, T92, P515, T515, and P79 on KL47 strains, and T444, P444, P24, and P79 on KL52 strains (Supplementary Table S2). Three phages demonstrated a host range of three different capsule types; P1051 and S419 were active against KL3, KL6, and KL10, while phage P79 was active against KL10, KL47, and KL52 strains. Nine phages that could infect different capsular types of MDR *A. baumannii* were selected for further study based on the results of the host range determination. 

### 2.3. Phage Adsorption

**Figure 3:**
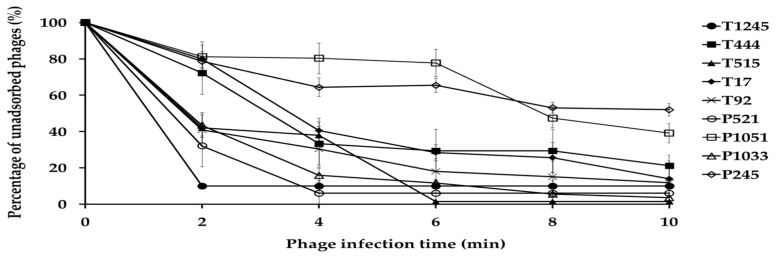
Adsorption of nine phages to their primary bacterial hosts. The free phage proportion was the amount of non-adsorbed phage to the amount of phage used for infection. Three independent experiments were performed in triplicate and error bars represent the standard deviation.

The adsorption assay was used to evaluate the ability of phages to bind to primary host bacterial cells (Figure 3). Within 10 min of the initiation of infection, the adsorption rates for phage T1245, T444, T515, T17, T92, P521, and P1033 ranged from 79 to 99%. Approximately 50% of the phage particles adsorbed to *A. baumannii* strains were observed for P1051 and P245 at 8 min post-infection. 

### 2.4. Life Cycle of Phage

**Figure 4:**
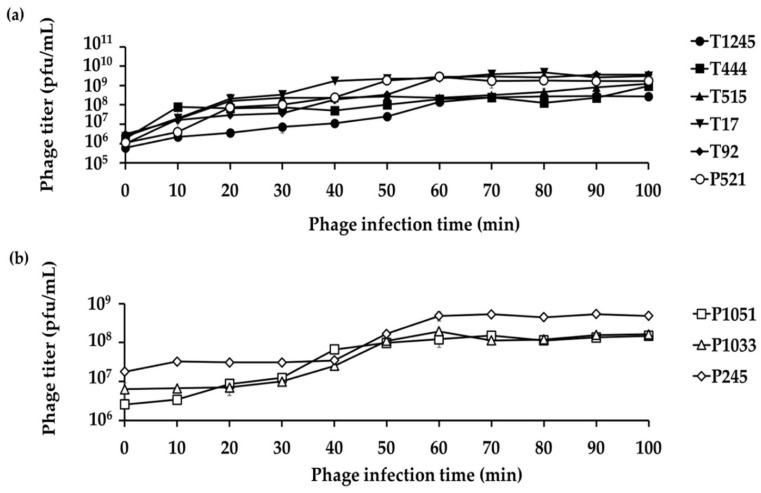
A single growth cycle of nine representative phages on their primary host strains. (**a**) Six phages showed a short latent period (2–6 min) and large burst size between 396 and 531 phage particles per infected host cell. (**b**) Three phages with a longer latent period (10–40 min) had an average burst size of 17–38 phage particles per infected host cell. Three independent experiments were performed in triplicate and error bars represent the standard deviation.

A single growth cycle (Figure 4) was conducted with the primary host strain to determine the latent period and burst size of individual phages, which are important characteristics of the phage infection process. The analysis showed a latent period of 2, 6, 2, 4, 6, and 4 min for six phages (T1245, T444, T515, T17, T92, and P521, respectively) (Supplementary Figure S2) and 10, 20, and 40 min for three phages (P1051, P1033, and P245, respectively) (Figure 4b). The average burst sizes were 396 to 531 phage particles per infected host cell for T1245, T444, T515, T17, T92, and P521 (results not shown). The three phages with the longer latent periods had an average burst size of 17 to 38 phage particles per infected host cell. 

### 2.5. Stability Tests

**Figure 5:**
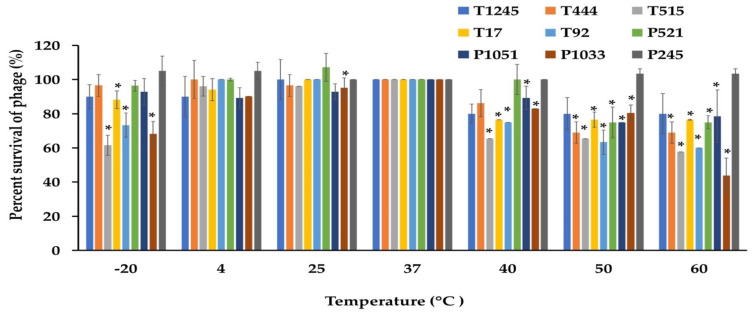
The stability of nine phages at various temperatures was measured for 2 h. The results were calculated as the percentage of surviving phage. Three independent experiments were performed in triplicate, and error bars represent the standard deviations (*, *p* < 0.05).

**Figure 6:**
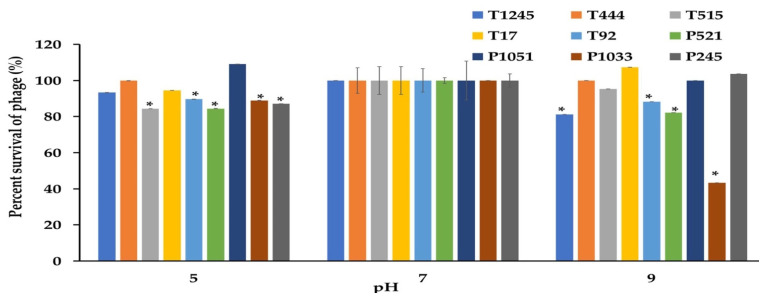
The stability of nine phages at various pHs was measured for 2 h. The results were calculated as the percentage of surviving phage. Three independent experiments were performed in triplicate, and error bars represent the standard deviations (*, *p* < 0.05).

The desirable characteristics of candidate phages for therapeutic applications should include stability under different conditions of temperature and pH as well as the ability to maintain infectivity after long-term storage. The phage stability was investigated at different temperatures and pH conditions. As shown in Figure 5, most phages were stable at temperatures ranging from −20 to 60 °C when exposed for 2 h. At 60 °C, a significant decrease of more than >50% in the survival rate was observed for phage P1033 when compared with the control at 37 °C. However, no phage showed lytic activity after incubation at 70 °C. Most phages showed greater than 80% viability over a broad pH range (5.0 to 9.0) after incubation for 2 h. However, 47.6% survival was observed for phage P1033 at pH 9 (Figure 6). The results suggested that the infectivity of the phage remained intact when exposed to broad temperature and pH conditions. Stock titers were stable for at least two years in SM buffer at 4 °C. However, a slight decrease was observed in titers for some phages (Table S1). 

### 2.6. Genome Annotation and Analysis

**Figure 7:**
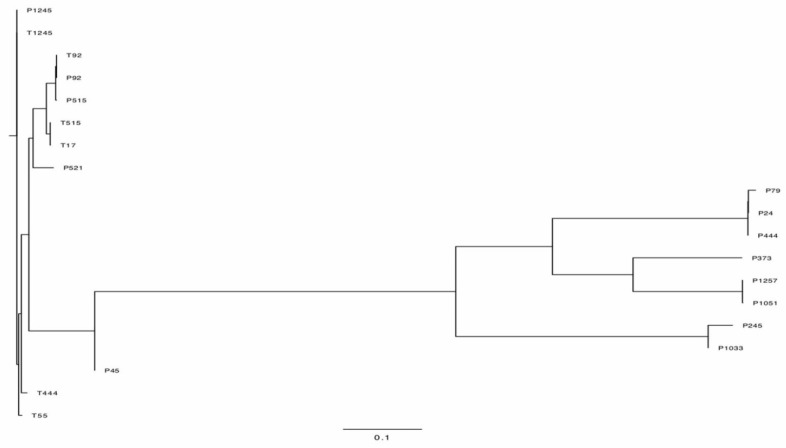
Phylogenetic tree showing the relatedness of phages used in this study.

**Figure 8:**
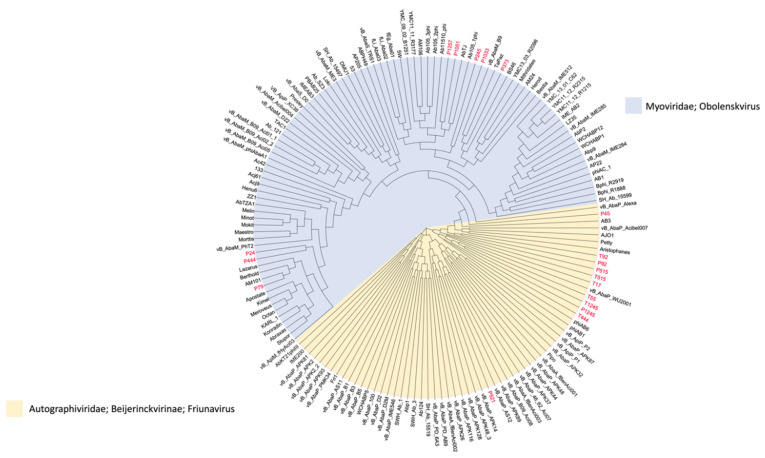
Whole-genome neighbor-joining phylogeny inferred from MASH distances of assemblies in the GenBank data set. The isolated phages used in this study are in red color, the family/genus of each phage is represented as a colored background, and a tab-delimited matrix of the MASH distances between each pair of isolates is presented as supplementary data.

The complete genome sequences were determined by high-throughput sequencing. The phylogenetic tree figure represents the DNA relatedness among nineteen phages based on whole-genome sequences (Figure 7) and the distance matrix between two DNA sequences (Supplementary Table S3). The dendrogram for nineteen phages was drawn based on sequence comparison with 138 *Acinetobacter* Caudvirales phages in GenBank assemblies to examine the distribution of *A. baumannii* phages (Figure 8) and a tab-delimited matrix of the MASH distances between each pair of isolates was presented as supplementary data (Supplementary Table S4). The results from taxonomy-based phylogeny, together with TEM images (Supplementary Figure S3), confirmed that eight phages (P444, P24, P105, P1033, P245, P1257, P373, and P79) and eleven phages (P1245, T1245, T444, P515, T515, T55, T17, P92, T92, P521, and P45) were designated to the Myoviridae and Podoviridae (Autographivirinae subfamily) family, respectively, as members of order Caudovirales, following the current guidelines of the ICTV (International Committee on Taxonomy of Viruses). Phages with long and contractile tails are grouped in the family Myoviridae, while those with short tails are in the Podoviridae family. Eight phages (T1245, T444, T515, T17, T92, P521, P1033, and P245) were selected for further study as the results of the host range determination (phages against different KL strains) and genomes have been fully sequenced. Based on life cycle, phages were grouped as virulent or temperate. Six of the phages were classified as lytic or virulent, including T1245, T444, T515, T17, T92, and P521. According to the turbid plaques and the presence of integrase genes in their genomes, P1033 and P245 were considered as temperate phages. The analysis of the whole-genome sequence revealed that seven of the eight phages (T1245, T444, T515, T17, T92, P521, and P245) had a genome length between 40 and 50 kb, while P1033 had a genome of 136 kb. The genome of phages had an average G + C content of 38–39% and showed a similar pattern of 39% of the host’s average genome. The correlation between the host and phage genomic G + C content is involved in phage and host evolution, indicating that they occurred during the same period. It is now accepted that horizontal gene transfer regularly occurs between bacteriophages and their hosts. This study is similar to the 39.15% G + C content reported for the genome of Abp1 (GenBank Acc. No. NC_021316), a lytic phage against *A. baumannii*. Phages T1245, T444, T515, T717, T92, and P521 displayed more than 90% homology to four phages belonging to the Autographiviridae subfamily, including *Acinetobacter* IME200 (GenBank Acc. No. NC_028987), *Acinetobacter* phiAB1 (GenBank Acc. No. NC_028675), *Acinetobacter* vB_AbaP_AS12 (GenBank Acc. No. NC_041914), and *Acinetobacter* phage Fri1 (GenBank Acc. No. NC_028848.1). A high percentage of the genomes of the sequenced phages, 80–90%, are represented by coding sequences (CDSs). According to the identification of coding sequences, the six lytic phages consisted of 47–54 CDSs, and 49–63% of genes were predicted as putative functional proteins. The genomes of phage P245 and P1033 were found to contain 59 and 180 CDSs, respectively, of which 28% and 24% of genes were predicted to encode proteins of known function, respectively (Supplementary Table S5). The known functions of CDSs encoding proteins were divided into several groups, including structure and morphogenesis, tRNA nucleotidyltransferase, DNA synthesis, regulation and replication, lysis, and homing endonuclease (Supplementary Table S6). Among these phages, no gene associated with antibiotic resistance or toxin production was found. The commonly presented genes in more than 50% of phages in this study are presented in Table 1. The results of the conserved sequence segments by a progressive Mauve alignment indicated that most of the protein functions were similar within six phages (T1245, T515, T17, T92, P521, and T444) and two phages (P1033 and P245), but the CDSs organization and direction were different (Supplementary Figure S4). 

## 3. Discussion

The global prevalence of drug resistance in clinical isolates of *A. baumannii* is a public health concern. Therefore, alternative strategies to treat infections caused by MDR *A. baumannii* are urgently needed. Phage therapy, the use of bacteriophage viruses to treat bacterial infections, is widely being reconsidered as an alternative to antibiotics. The host range of a bacteriophage is an important feature to be considered in the selection of phages for potential therapeutic applications. The range of bacterial hosts that can be infected by an individual phage may vary considerably and some phages, such as those active against *Klebsiella* spp, may infect bacteria of different genera [23,24]. In contrast, phages against *A. baumannii* have been reported to infect only *Acinetobacter* spp. [25]. A limited host range is beneficial in restricting the killing of other bacterial species and preventing the disruption of the microbiota. The narrow host specificity of phage could mitigate the development of resistance, which is an advantage over antibiotics [26]. Previous studies revealed that two *A. baumannii* lytic phages belonging to Myovirus and Podovirus were able to propagate on approximately 53% (15 of 28) of tested strains [27]. In this study, phages were isolated against five common capsular types of *A. baumannii*. Lytic phages including T1245, T444, and T515 could infect all strains of their host KL type. None of the isolated phages in this study were active against *A. baumannii* KL2 and KL49 capsule types, indicating the absence of phage-specific receptors on host cell surfaces, which predominantly influences the host range of phages. Within 10 min of the initiation of infection, the adsorption rate for phages T1245, T444, and T515 ranged from 80 to 99%. These phages had larger burst sizes with shorter latent periods, compared with previously isolated *Acinetobacter* phages of the order Caudovirales [27]. Furthermore, the selected phages are relatively stable over a wide range of pH and temperatures, which are important in both storage conditions and transportation for use in a clinical scenario. The genome sequences of three lytic phages (T1245, T444, and T515) showed more than 90% homology to other genomes annotated as *Acinetobacter* phages, including IME200, phiAB1, vB_AbaP_AS12, and Fri1. Phages T1245, T444, and T515 have evolved various virion-associated proteins with depolymerase activity and endolysin. Bacteriophages have specific recognition for receptors located on the bacterial cell surface, such as capsular polysaccharides and extracellular polysaccharides. The presence of depolymerases is described and characterized in the phage virion within the tail fibers or tailspikes. The genes encoding tail fiber proteins were presented in all the selected *A. baumannii* phages. Phage-encoded capsule depolymerases from a tail fiber protein of IME200 was previously reported [28]. Certain studies have represented the relationship of these genes with the capsular polysaccharides function as primary receptors on the bacterial surface [29,30,31]. Moreover, in this study, the genome sequences of three lytic phages disclosed no protein toxin-coding genes.

As natural killers of bacteria, phages are recognized as promising alternatives to antibiotics in treating pulmonary bacterial infections. Phage therapy has been applied in personalized therapy in the treatment of CRAB in a patient with chronic obstructive pulmonary disease [32]. The development of phage-based therapy combined with antibiotics has been demonstrated to enhance the antibacterial effects in *Gallerai mellonella* models [33,34]. Recently, the use of phage cocktails with antibiotics resulted in the reduction in *A. baumannii* biofilm in human urine models [35]. Due to the ability of phage-derived systems to integrate plasmid DNA into the host’s chromosomes, phages containing the integrase gene can be used as a viral vector-mediated gene therapy [36]; therefore, the temperate phages in this study (P1033 and P245) could be candidates. Moreover, phage therapy could be used in secondary *A. baumannii* infections in critical coronavirus disease (COVID-19) patients with antibiotic treatment failure [37]. However, further evaluation in well-designed clinical trials is required to confirm the potential of phage in future studies.

## 4. Materials and Methods

### 4.1. Bacterial Strains and Culture Conditions

Forty-nine multidrug-resistant *A. baumannii* isolates from patients at Songklanagarind, Siriraj, and Thammasat hospitals with different sample sources, including saliva (37 isolates), blood (6), and tissue (6), were used for phage characterization [6]. All isolates belonged to multilocus sequence type (MLST) 2 (ST2) and expressed the common capsule types KL2 (4 isolates), KL3 (2), KL6 (25), KL10 (8), KL47 (2), KL49 (5), and KL52 (3). All bacterial cultures were tested for viability and purity by sub-culturing on tryptic soy agar (TSA). Bacterial stocks were prepared with tryptic soy broth (TSB) with 20% glycerol and kept at −80 °C for further investigations.

### 4.2. Isolation of Bacteriophages

The protocol described by Clokie and Kropinski (2009) was used for phage isolation, with minor modifications [38]. Briefly, sewage water samples were collected from hospitals in Thailand. Ten milliters of sewage water sample was mixed with 0.3 g of TSB powder and then inoculated with 100 μL of overnight bacterial culture. After overnight incubation at 37 °C with continuous shaking, the mixture was centrifuged at 5000× *g* for 20 min. The supernatant was filtered through a 0.45 μm membrane filter (Millipore) and kept at 4 °C until use. An agar overlay method was used for the examination of bacteriophage activity; an overnight bacterial culture was mixed with molten soft nutrient agar (0.75% agar) and immediately poured onto a 1.5% agar plate. Ten milliters of supernatant was dropped onto the plate, and the plate was incubated overnight at 37 °C.

### 4.3. Purification of Bacteriophages

Single plaques were picked from the plate with a sterile pipette tip, transferred to sterile SM buffer (100 mM NaCl, 8 mM MgSO_4_ • 7H_2_O, 50 mM Tris-Cl; pH 7.5), and stored at 4 °C. The solution was diluted tenfold in SM buffer and mixed with TSB containing 200 μL of log-phase bacterial culture at an optical density (OD)_600_ of 0.8–0.9. An agar overlay method was used for the purification and determination of the phage titer [39]. The single plaque isolation procedure was repeated three times to obtain purified bacteriophage.

### 4.4. Phage Lysate Preparation

The plate containing purified phage with a high phage titer was mixed with 5 mL of SM buffer and incubated overnight at 4 °C. The supernatant was collected and centrifuged at 5000× *g* for 30 min at 4 °C. The pellet was discarded and the phage lysate was filtered through a sterile 0.45 μm filter. Subsequently, 1 μL/mL of chloroform was added and the bacteriophage stock was kept at 4 °C until used.

### 4.5. Determination of Phage Host Range

The host range of each phage was determined by spotting onto each of the 49 *A. baumannii* as described previously [40]. In brief, a TSA plate was overlaid with molten soft nutrient agar (0.8% agar) containing 200 μL of each log-phase *A. baumannii* isolate and spotted with 10 μL of phage lysate. Plates were dried and incubated at 37 °C to assess the lytic activity.

### 4.6. Phage Adsorption

The adsorption of phages to the bacterial host was examined as described by Hua et al. (2018) [41], with minor modifications. Exponentially grown *A. baumannii* was adjusted to 10^8^ cfu/mL and mixed with phage lysate at an MOI of 0.01. After incubation at 37 °C for 10 min, 100 μL of sample was added into 900 μL of cold TSB, centrifuged at 13,000× *g* for 5 min to eliminate debris, and the supernatant was filtered through a sterile 0.45 μm membrane filter. The supernatants were used for plaque assays to determine the titers of the non-adsorbed phages. Adsorption curves were constructed based on the percentage of non-adsorbed phages at different time intervals. Each experiment was performed in triplicate.

### 4.7. One-Step Growth Parameters

The phage growth characteristics (latent period and burst size) were determined as described by Drulis-Kawa et al. (2011) [42], with some modifications. Briefly, the exponential phase *A. baumannii* cultures were adjusted to 10^8^ cfu/mL and incubated with phage lysate at a multiplicity of infection (MOI) of 0.001. A bacteriophage was allowed to adsorb to the bacterial surface at 37 °C for 10 min prior to determine the latency period. The mixture was centrifuged and the pellet was washed twice in 10 mL of LB broth. The samples were collected and the soft agar overlay method was used to determine the phage titer. The time interval between initiation of phage infection and release of progeny was defined as the latent period. The ratio of phage titer to the number of infected bacterial cells for one cycle was calculated as the burst size. Each experiment was performed in triplicate.

### 4.8. Sensitivity of Phage Particles to Temperature and pH

The thermal and pH stabilities of phages were examined according to the method of Yang et al. (2019) [43]. Briefly, 10^5^ pfu/mL of phages were incubated at different temperatures (−20, 4, 25, 37, 40, 50, 60, and 70 °C), and at various pH values (3, 5, 7, 9, and 11). For both experiments, samples were taken at 2 h and the surviving phage were quantified by titration. Phage incubated at 37 °C and pH 7 were used as the control for the temperature and pH variation experiments, respectively. Each experiment was performed in triplicate. Statistical analysis between the test and control sets were performed by an unpaired *t*-test using GraphPad Prism v8, *p* < 0.05 for significance. 

### 4.9. Phage Stock Stability

The titer of each phage stock was prepared from a 10-fold dilution in SM buffer and determined by the double agar overlay method after preparation of the stock. Stock titers were also determined after two years storage at 4 °C.

### 4.10. Transmission Electron Microscopy (TEM)

The phage morphology was determined by TEM. Purified phage particles (10^7−10^ pfu/mL) were allowed to adsorb to formvar-coated 200-mesh copper grids for 10 min and washed twice for 1 min in double-distilled water (ddH_2_O). The particles were negatively stained with 2% (wt/vol) uranyl acetate for 5 min and allowed to air dry. The samples were observed under TEM (JEOL, JEM-2010).

### 4.11. Extraction of Genomic DNA 

The phage lysate was precipitated using 8% (wt/vol) polyethylene glycol 8000 (PEG 8000) and 1 M NaCl precipitation according to Sambrook and Russell (2001) [44]. The phage lysate was added with 0.9 mL of PEG/NaCl, and incubated overnight at 4 °C. The culture was centrifuged at 17,000× *g* for 20 min and the pellet was suspended in 0.5 mL of PBS (phosphate buffered saline; pH 7.4). The phage DNA was prepared by the Phase Lock Gel System (phenol–chloroform method) of Pickard (2009) [45]. Briefly, contaminants were removed by incubation with 1 mg/mL of DNase I and 12.5 mg/mL of RNase A for 30 min at 37 °C. The mixture was added to 20% SDS and 10 mg/mL of proteinase K and incubated for a further 30 min at 37 °C. The mixture was aliquoted into 500 μL volumes in 1.5 mL Phase Lock gel Eppendorf tubes and extracted with 0.5 mL of phenol:chloroform:isoamyl alcohol (IAA) (25:24:1), followed by centrifugation at 5000× *g* for 5 min in order to separate the phases. The top aqueous phase was transferred into a fresh Phase-lock gel tube and the above step was repeated, chloroform:isoamyl alcohol (24:1) was added, and the mixture was centrifuged at 5000× *g* for 5 min. To precipitate phage DNA, the aqueous phase was transferred into a 1.5 mL Eppendorf tube, and then 45 μL of 3 M sodium acetate (pH 5.2) and two volumes of absolute ethanol were added to the mixture and incubated at room temperature for 20 min. The precipitated DNA was collected by centrifugation at 17,000× *g* for 20 min, washed twice with 70% ethanol, and air dried. The DNA pellet was suspended to a final volume of 30 μL with TE buffer (10 mM Tris HCl, 1.0 mM EDTA). 

### 4.12. Whole Genome Sequencing, Assembly, and Genome Annotation

Approximately 0.5 μg of each DNA preparation was sequenced using the Illumina HiSeq X10 platform at the Wellcome Sanger Institute (WSI). A high-throughput pipeline was employed to assemble Illumina sequencing reads into contigs [46]. This pipeline relies on Velvet v1.2 [47], VelvetOptimiser v2.2.5 (http://github.com/tseemann/VelvetOptimister (accessed on 15 November 2019)), SSPACE [48], and GapFiller [49]. The assemblies were annotated using Prokka v1.5 [50] and a genus-specific database [51]. All of the software developed by Pathogen Informatics at the WSI is freely available for download from GitHub (http://github.com/sanger-pathogens/assembly_improvement (accessed on 15 November 2019)) under an open-source license, GNU GPL 3. The improvement step of the pipeline is also available as a standalone Perl module from CPAN (http://search.cpan.org/~ajpage/ (accessed on 15 November 2019)).

### 4.13. Bioinformatics Analysis

The assembled whole-genome sequences were searched and compared with other phages for similarities between biological sequences using the Basic Local Alignment Search Tool (BLASTX) (https://blast.ncbi.nlm.nih.gov/Blast.cgi (accessed on 5 February 2021)). The assemblies were visualized, aligned, and compared using MAUVE software ver. 2.3.1 [52] (https://darlinglab.org/mauve/mauve.html (accessed on 3 September 2021)). A phylogeny of phage sequences based on MASH distances was constructed using MASHTREE by the neighbour-joining method [53] (https://github.com/lskatz/mashtree (accessed on 6 January 2022)). Phylogenetic trees between DNA relatedness of 19 phages were visualized using Figtree V1.4.3 (http://tree.bio.ed.ac.uk/software/figtree/ (accessed on 6 January 2022)). A pangenome analysis using Roary V3.11.2 was performed with BLASTP > 90% sequence identity for clustering (https://www.sanger.ac.uk/tool/roary/ (accessed on 6 January 2022)).

### 4.14. Accession Number

The whole-genome sequencing data generated in this study have been deposited into the European Nucleotide Archive (ENA; https://www.ebi.ac.uk/ena (accessed on 21 January 2022)) under study accession number PRJEB33567. Sample accession numbers for each sequenced bacteriophage are listed in Supplementary Table S7.

## 5. Conclusions

The gene-encoded depolymerase and endolysin identified in the newly isolated lytic *A. baumannii* phages, together with the absence of virulence-associated genes, render further studies for potential applications. Moreover, these lytic phages demonstrated a short generation and adsorption time, high degree of host specificity, ability to produce high numbers of virions, and physical stability over a wide range of temperature, pH, and storage conditions. 

## Figures and Tables

**Table 1:** The common genes present in more than 50% of phages in this study.

Genes
Structural protein
DNA primase/helicase
EF hand domain protein
Holin
Phage capsid and scaffold protein
Putative DNA exonuclease
Putative DNA helicase
Hypothetical protein
Head-to-tail joining protein
Metallo-dependent phosphoesterase
Putative DNA endonuclease VII
Putative DNA maturase B
Capsid and scaffold protein
DNA polymerase I

## Data Availability

Data is contained within the article and Supplementary Material.

## Supplementary Materials

The following supporting information can be downloaded at: https://www.mdpi.com/article/10.3390/ph15040443/s1, Table S1: Isolated phage. A total of 20 phages against multidrug-resistant isolates of *Acinetobacter baumannii*; Table S2A: Cross-infectivity of the isolated bacteriophages; Table S2B: Summary table of phage infectivity on different KL Types; Table S3: Corresponding matrix of the DNA distances; Table S4: A tab-delimited matrix of MASH distances between each pair of isolates; Table S5: The list of CDSs/gene in the genome of phages and their putative functions; Table S6: The common presence of genes in study; Table S7: Sample accession numbers of sequenced bacteriophages; Figure S1: The presence of phage-encoded polysaccharide depolymerases; Figure S2: A single growth cycle of six phages showed a short latent period (2-6 min); Figure S3: Morphology of T444 (a) and P1033 (b) by transmission electron microscopy; Figure S4: The genome organization of eight phage and reference Acinetobacter phage IME200 were compared using a progressive Mauve progressive alignment.
