# Supplementary material for: Isolation and Characterisation of Bacteriophage Selective for Key Acinetobacter baumannii Capsule Chemotypes
Source: Pharmaceuticals (Basel). 2022 Apr 2;15(4):443. doi: 10.3390/ph15040443 (PMC9027227; doi:10.3390/ph15040443)
Supplement: Supplementary file 1 [file pharmaceuticals-15-00443-s001.zip › Supplemental Material 2.pdf]

## SUPPLEMENTAL MATERIAL

**TABLE S1.** Isolated phage. A total of 20 phages against multidrug-resistant isolates of *Acinetobacter baumannii.*

| Phage | Host | Plaque morphology | Clear zone (mm) | Titer | Titer after one yaer |
|---|---|---|---|---|---|
| P1245 | ABMYH-1245 | medium-size, bright | 4 | $10^{10}$ | $10^{10}$ |
| T1245 | ABMYH-1245 | big, bright | 10 | $10^{10}$ | $10^{10}$ |
| P444 | ABMSP-444 | extremely small, slightly dim | 0.5-1 | $10^{3}$ | $10^{3}$ |
| T444 | ABMSP-444 | big, bright | 10 | $10^{10}$ | $10^{9}$ |
| P24 | ABMSP-444 | extremely small, slightly dim | 0.5-1 | $10^{3}$ | $10^{3}$ |
| P515 | ABMASP-515 | big, bright | 8-9 | $10^{7}$ | $10^{6}$ |
| T515 | ABMASP-515 | big, bright | 8-9 | $10^{10}$ | $10^{9}$ |
| T55 | ABAPSP-55 | big, bright | 8-9 | $10^{9}$ | $8x10^{7}$ |
| T17 | ABJNSP-17 | big, bright | 8 | $10^{10}$ | $10^{10}$ |
| P92 | ABJNH-92 | big, bright | 8-9 | $10^{10}$ | $10^{10}$ |
| T92 | ABJNH-92 | big, bright | 8-9 | $10^{10}$ | $10^{10}$ |
| P521 | ABMASP-521 | medium-size, bright | 7 | $10^{11}$ | $10^{10}$ |
| P1051 | ABMYH-1051 | small, slightly dim | 2 | $10^{7}$ | $10^{7}$ |
| P1033 | ABMYH-1033 | small, slightly dim | 2 | $10^{7}$ | $10^{7}$ |
| P245 | ABMYSP-245 | small, slightly dim | 3 | $10^{6}$ | $10^{5}$ |
| S419 | ABMYSP- 419 | medium-size, bright | 4 | $10^{7}$ | $10^{7}$ |
| P45 | AB45 | big, bright | 8-9 | $10^{7}$ | $10^{7}$ |
| P1257 | ABMYH -1257 | small, slightly dim | 2 | $10^{8}$ | $10^{6}$ |
| P79 | AB79 | extremely small, slightly dim | 0.5-1 | $10^{3}$ | $10^{3}$ |
| P373 | ABMASP-373 | small, slightly dim | 2 | $10^{4}$ | $10^{4}$ |

**(a)** **(b)** **(c)**

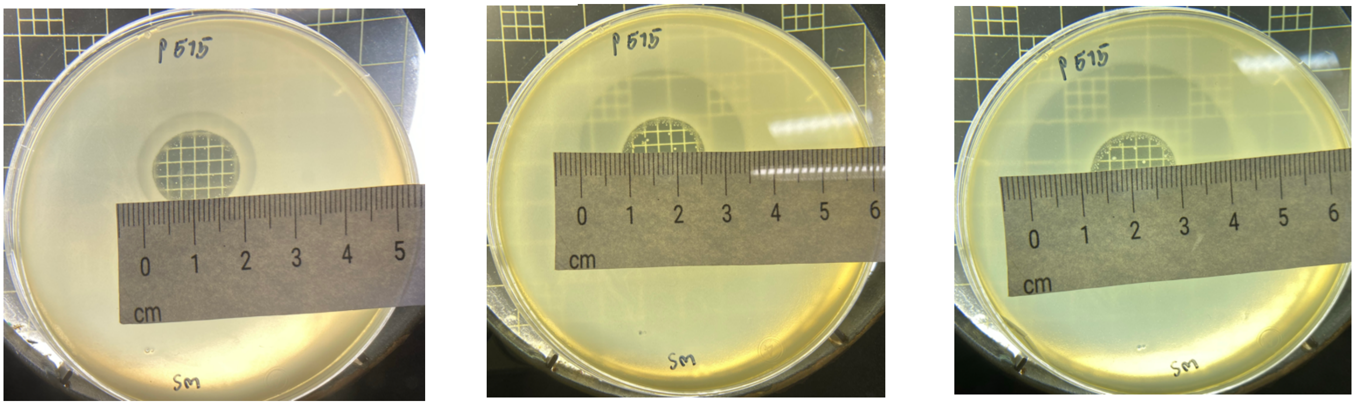


**FIGURE S1.** The presence of phage-encoded polysaccharide depolymerases. The plaques were surrounded by a translucent halo, which increased in diameter over time of incubation while the plaque size remains constant. Incubation time of **(a)** 24h; **(b)** 48h; **(c)** 72h at 37°C.

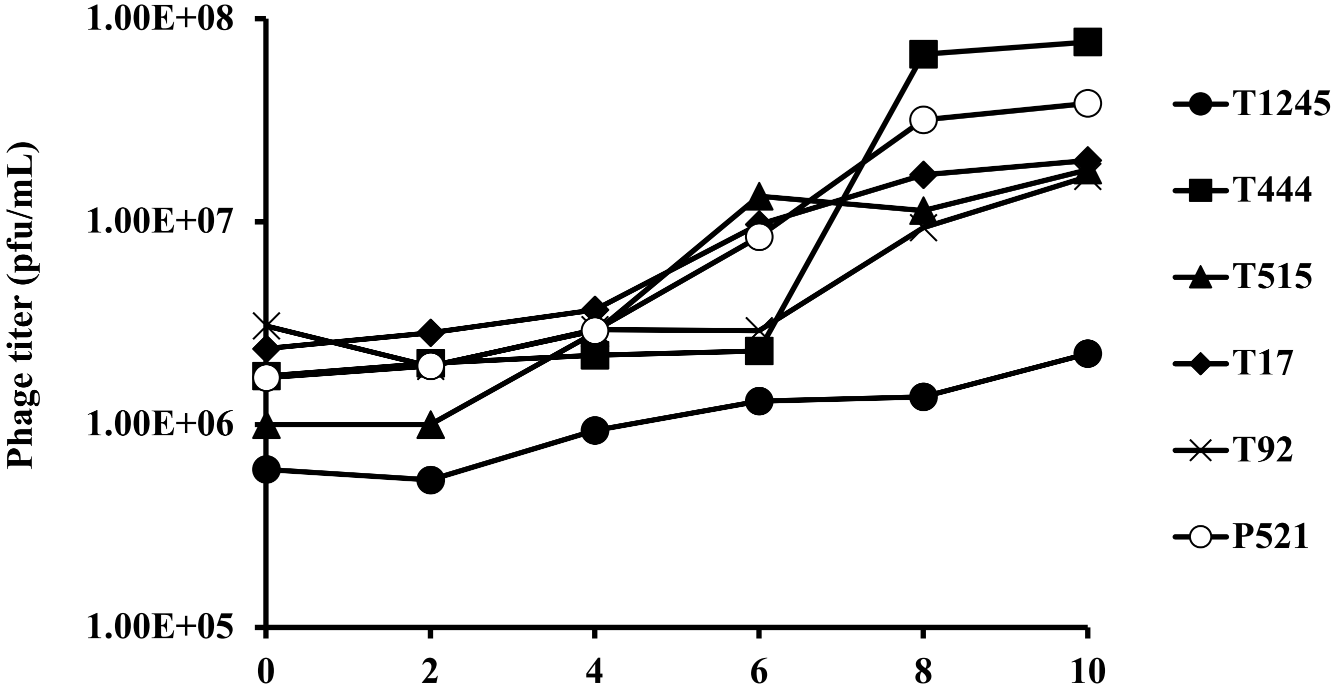


**FIGURE S2.** A single growth cycle of six phages showed a short latent period (2-6 min).

(a) (b)

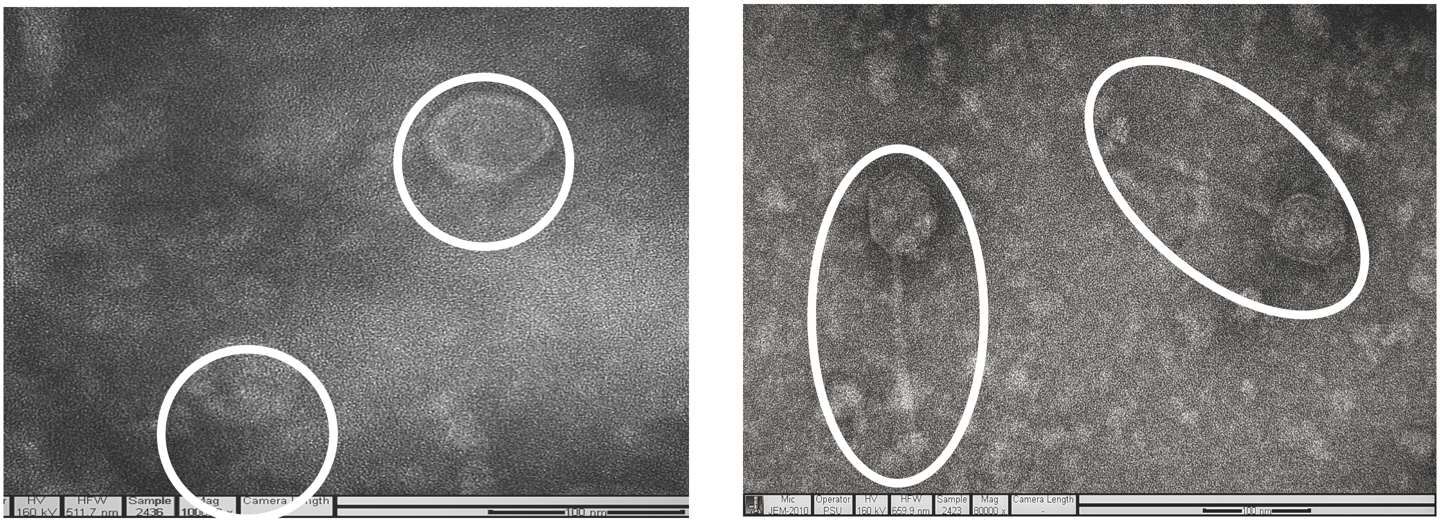


**FIGURE S3.** Morphology of T444 **(a)** and P1033 **(b)** by transmission electron microscopy. T444 possessed an icosahedral head of 51.09 nm in diameter and very short, which was nearly invisible. P1033 possessed an icosahedral head of 64.34 nm in diameter and non-contractile tail of 156.03 nm in length and 9.56 nm in width. Thus, T444 and P1033 were designated to the family Podoviridae and Myoviridae in the order of Caudovirales, respectively, following the current guidelines of the ICTV (International Committee on Taxonomy of Viruses). The scale bar represents 100 nm. Magnification: × 100,000.

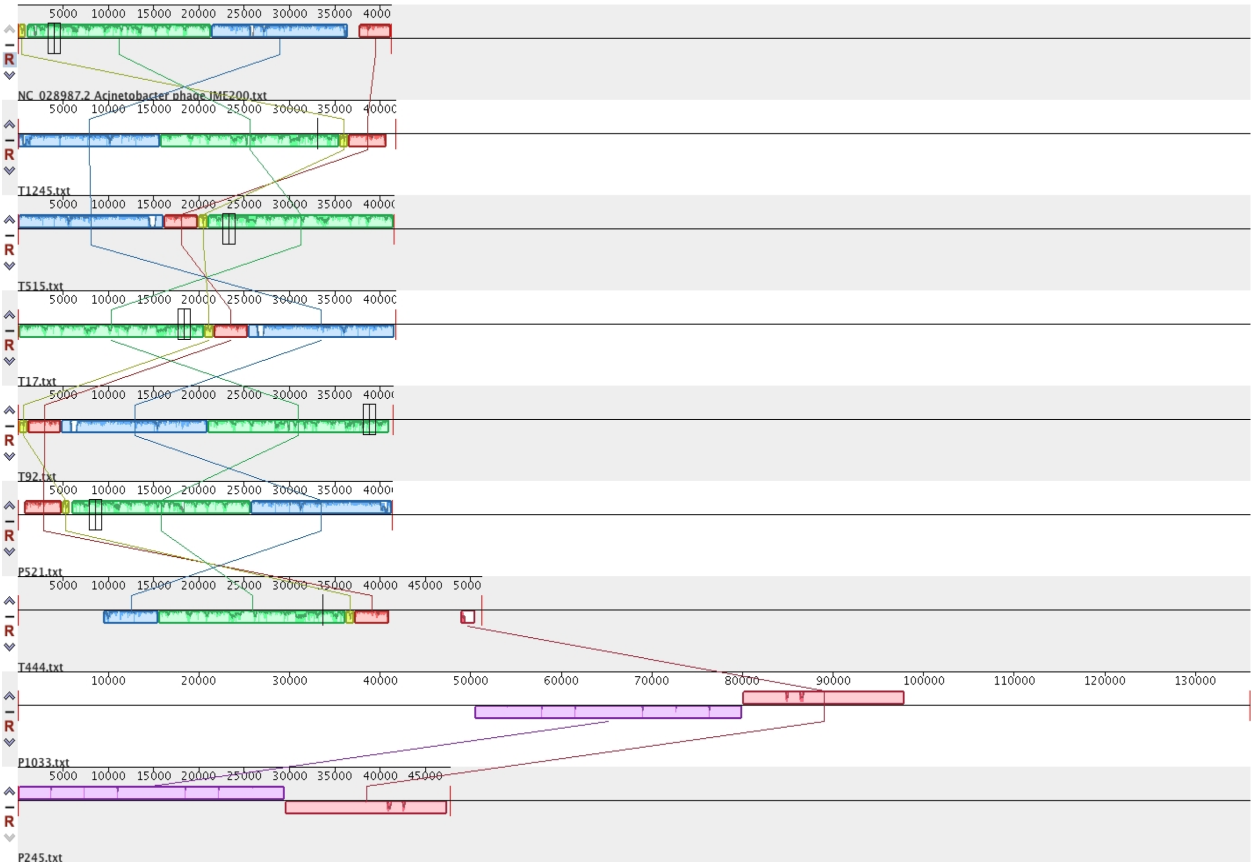


**FIGURE S4.** The genome organization of eight phage and reference *Acinetobacte***r** phage IME200 were compared using a progressive Mauve progressive alignment. There are three Panels of each alignment, including the base pair scale of each genome, the colored segments indicate the genome composition of each phage (the conserved local collinear blocks), and the the name of each phage. The connecting-coloured lines show the similar DNA regions between each phage genome. Within each block a similarity profile of the genome sequence is displayed. The height of the similarity profile corresponds to the average level of conservation in that region of the genome sequence.
